# Characterization of the Gene Encoding S-adenosyl-L-methionine (AdoMet) Synthetase in *Penicillium chrysogenum*; Role in Secondary Metabolism and Penicillin Production

**DOI:** 10.3390/microorganisms10010078

**Published:** 2021-12-30

**Authors:** Rebeca Domínguez-Santos, Katarina Kosalková, Isabel-Clara Sánchez-Orejas, Carlos Barreiro, Yolanda Pérez-Pertejo, Rosa M. Reguera, Rafael Balaña-Fouce, Carlos García-Estrada

**Affiliations:** 1 INBIOTEC (Instituto de Biotecnología de León), Av. Real 1, 24006 Leon, Spain; Rebeca.Dominguez@uv.es (R.D.-S.); kkos@unileon.es (K.K.); csanchez@inbiotec.com (I.-C.S.-O.); c.barreiro@unileon.es (C.B.); 2 Área de Bioquímica y Biología Molecular, Departamento de Biología Molecular, Campus de Vegazana, Universidad de Leon, 24007 Leon, Spain; 3 Departamento de Ciencias Biomédicas, Campus de Vegazana, Universidad de Leon, 24007 Leon, Spain; myperp@unileon.es (Y.P.-P.); rmregt@unileon.es (R.M.R.); rbalf@unileon.es (R.B.-F.)

**Keywords:** S-adenosyl-L-methionine, polyamines, penicillin, secondary metabolism, *Penicillium chrysogenum*

## Abstract

The filamentous fungus *Penicillium chrysogenum* (recently reidentified as *Penicillium rubens*) is used in the industrial production of the β-lactam antibiotic penicillin. There are several mechanisms regulating the production of this antibiotic, acting both at the genetic and epigenetic levels, the latter including the modification of chromatin by methyltransferases. S-adenosyl-L-methionine (AdoMet) is the main donor of methyl groups for methyltransferases. In addition, it also acts as a donor of aminopropyl groups during the biosynthesis of polyamines. AdoMet is synthesized from L-methionine and ATP by AdoMet-synthetase. In silico analysis of the *P. chrysogenum* genome revealed the presence of a single gene (Pc16g04380) encoding a putative protein with high similarity to well-known AdoMet-synthetases. Due to the essential nature of this gene, functional analysis was carried out using RNAi-mediated silencing techniques. Knock-down transformants exhibited a decrease in AdoMet, S-adenosyl-L-homocysteine (AdoHcy), spermidine and benzylpenicillin levels, whereas they accumulated a yellow-orange pigment in submerged cultures. On the other hand, overexpression led to reduced levels of benzylpenicillin, thereby suggesting that the AdoMet synthetase, in addition to participate in primary metabolism, also controls secondary metabolism in *P. chrysogenum*.

## 1. Introduction

**Figure 1:**
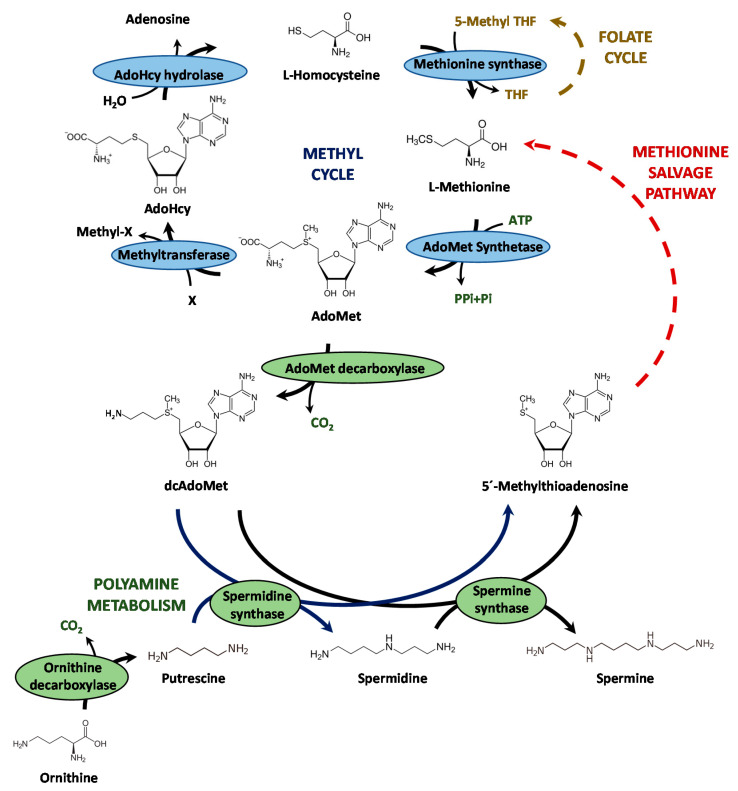
Schematic representation of the methyl cycle and the polyamine biosynthetic pathway. AdoMet: S-adenosyl-L-methionine; AdoHcy: S-adenosyl-L-homocysteine; dcAdoMet; decarboxylated AdoMet; THF: tetrahydrofolate.

The filamentous fungus *Penicillium chrysogenum*, re-identified as *Penicillium rubens* [1], is well known for being the microorganism used in the industrial production of the β-lactam antibiotic benzylpenicillin. The biosynthesis of this antibiotic is a well-characterized process that begins with the non-ribosomal condensation of α-aminoadipic acid, L-cysteine and L-valine to form L-δ(α-aminoadipyl)-L-cysteinyl-D-valine. Then, this step is followed by cyclization of the tripeptide to constitute the β-lactam ring structure, thus giving rise to isopenicillin N. The pathway is completed with the substitution of the α-aminoadipyl side chain of isopenicillin N with the CoA-activated form of phenylacetic acid (phenylacetyl-CoA), thereby constituting benzylpenicillin or penicillin G (for a recent review see [2]). The biosynthesis of benzylpenicillin is subjected to complex regulatory mechanisms, which act both genetically and epigenetically [3,4,5,6]. Epigenetic mechanisms acting on the modification of chromatin by acetyltransferases or methyltransferases exert an enormous influence on the expression of different secondary metabolism biosynthetic gene clusters [7]. LaeA is a global regulator of secondary metabolism that has a putative methyltransferase activity [8,9,10], which regulates penicillin biosynthesis in *P. chrysogenum* [5,11]. S-adenosyl-L-methionine (AdoMet) is the main donor of methyl groups for methyltransferases in transmethylation reactions in eukaryotic systems [12]. The biosynthesis of AdoMet (Figure 1) is catalyzed by AdoMet synthetase (EC 2.5.1.6) (also known as methionine adenosyltransferase, MAT) [13], which uses methionine and ATP as substrates in a reaction dependent on the presence of K^+^ and Mg^2+^ to yield AdoMet, pyrophosphate and inorganic phosphate [14]. In the next step, S-adenosyl-L-homocysteine (AdoHcy) is formed after the transfer of a methyl group from AdoMet and is further converted to adenosine and homocysteine by AdoHcy hydrolase [15]. The cycle is closed after the conversion of L-homocysteine into methionine (Figure 1). The AdoMet synthetase core structure is conserved between different organisms and include the motifs for L-methionine, ATP and PPi binding [16], and active-site catalytic amino acids [17]. In addition to being a universal donor of methyl groups, AdoMet is also a precursor for higher polyamines (spermidine and spermine), which play a crucial role in many physiological processes, including cell proliferation, gene expression and DNA recombination and repair [18]. For this, AdoMet is decarboxylated and releases aminopropyl moieties that are captured by putrescine and spermidine to synthesize spermidine and spermine, respectively [19] (Figure 1).

AdoMet and AdoMet synthetases have been well characterized in mammals and other eukaryotes [16,20,21,22], including *Saccharomyces cerevisiae* [23,24,25,26]. On the contrary, very little is known about their role in filamentous fungi, and the information available is the result of just three articles. In the first one, it was reported that variation in AdoMet levels in *Neurospora crassa* modifies the flux of AdoMet-dependent metabolic pathways [27]. In the second article, AdoMet synthetase of *Aspergillus nidulans* (AnSasA) was reported to regulate sporogenesis and the production of secondary metabolites [28]. Finally, and very recently, it was confirmed that in *Penicillium oxalicum* AdoMet synthetase (PoSasA) is essential for viability and for the expression of extracellular glycoside hydrolases (specifically cellulolytic enzymes) encoding genes [29]. Therefore, more information about AdoMet synthetases is necessary to determine the role of this enzyme and its product in filamentous fungi.

In this article we have performed an in silico analysis of the *P. chrysogenum* genome finding that this filamentous fungus contains a single-copy gene (Pc16g04380) that putatively encodes AdoMet synthase. Due to the essential nature of this gene, its functional analysis was carried out using RNAi-mediated gene silencing. The analysis of AdoMet, AdoHcy and the polyamines putrescine and spermidine levels in the knock-down transformants confirmed that Pc16g04380 encodes the *P. chrysogenum* AdoMet synthetase (PcSasA). Interestingly, our results indicate that AdoMet levels regulate secondary metabolism and penicillin biosynthesis in *P. chrysogenum*.

## 2. Materials and Methods

### 2.1. Strains, Media and Culture Conditions

*P. chrysogenum* Wisconsin 54-1255 is the reference strain for the genome sequencing project and has been re-identified as *P. rubens* [1]. For consistency with the traditional and well-recognized name, as well as with our previous works, we will use in this article *P. chrysogenum*. This fungus was grown in solid Power sporulation medium [30] for 7 days at 28 °C to obtain fresh-made sporulated plates. For expression analysis experiments, flasks cultures were carried out by inoculating fresh spores (approximately 10^8^) of *P. chrysogenum* in 100 mL of complex inoculum medium CIM [31] without phenylacetate. After incubation at 25 °C for 20 h in an orbital shaker (250 r.p.m.), aliquots (5%) were inoculated in CP complex penicillin production medium [31] in the absence or presence of 0.4% potassium phenylacetate, and incubated under the same conditions for up to 72 h. For other experiments, flask cultures were performed in a similar way, but using defined medium [30] for preinoculum, and defined medium with 0.1% potassium phenylacetate as final culture medium with a 10% inoculum.

*Escherichia coli* DH5α cells were used for plasmid amplification. Cells were grown in Luria–Bertani medium with ampicillin (100 μg/mL).

### 2.2. Plasmid Constructs

Plasmid pJL43-RNAi-SAMsil was constructed to give rise to Pc16g04380 knock-down transformants and was constructed as follows: Plasmid pJL43-RNAi [32] with the *ble* gene marker (for phleomycin resistance), was digested with *Nco*I in order to subclone a 467-bp fragment amplified with primers MatSilF (5′-CCATGGAGTAATCCTTTCCGGAG-3′) and MatSilR (5′-CCATGGTATCATGTTCGGCTATG-3′) from the third exon of the Pc16g04380 gene. This fragment was digested with *Nco*I (underlined in the primer sequences), thus yielding pJL43-RNAi-SAMsil.

Plasmid pOverSAM was used for the overexpression of the *P. chrysogenum* Pc16g04380 gene and was constructed as follows: Oligonucleotides MAToverF (5′-CACACCATGGATGGGCTCTGTTTC-3′) and MAToverR (5′- GCAGAAGGCCTTCAGAACTTGAGGG-3′) were used to amplify by PCR the 1317-bp Pc16g04380 gene. The amplicon was digested with *Nco*I and *Stu*I (underlined in the forward and reverse primer sequences, respectively) and was subcloned into pIBRC43 [33], previously digested with *NcoI*-*StuI*, and between the strong *Aspergillus awamori gdh* gene promoter and the *S. cerevisiae cyc1* transcriptional terminator.

Plasmid pJL43-Trp [34], which contains the *ble* gene as a selection marker under the control of the *gpdA* gene promoter and the trpC terminator, was used as helper plasmid in the transformations with pOverSAM, in order to allow further selection of overexpression transformants in the presence of phleomycin.

### 2.3. Transformation of P. chrysogenum Protoplasts, Extraction of Genomic DNA and Southern Blotting

*P. chrysogenum* protoplasts were obtained and transformed following standard laboratory protocols, which have been described elsewhere [35]. Upon transformation, protoplasts were grown in Czapek minimal medium [31], and the selection of positive clones was carried out by addition of phleomycin (final concentration 30 μg/mL) to Czapek medium.

Isolation of genomic DNA from *P. chrysogenum* and Southern blotting hybridization have been previously described [36,37].

### 2.4. RNA Extraction and RT-PCR Experiments

RNA was extracted from samples taken at 48 h from cultures of *P. chrysogenum* grown in complex medium (see above) using “RNeasy Mini Kit” columns (Qiagen, Hilden, Germany), following the manufacturer’s instructions. Total RNA was treated with RNase-Free DNase (Qiagen, Hilden, Germany), following the manufacturer’s instructions and quantified using a NanoDrop ND-1000 spectrophotometer. Prior to reverse transcription, the absence of contaminant DNA in the samples was tested by PCR.

For RT-PCR experiments, 200 ng of total RNA were retrotranscribed and amplified using the “SuperScript One-Step RT-PCR with Platinum Taq” system (Invitrogen Corporation), following the manufacturer’s instructions. MatSilF and MatSilR primers (see above) were used to assess Pc16g04380 expression levels. Expression of the γ-actin gene was used as control. For this purpose, primers RTactF (5′-CTGGCCGTGATCTGACCGACTAC-3′) and RTactR (5′-GGGGGAGCGATGATCTTGACCT-3′) were used. Quantitation (densitometry) of the intensity of the bands amplified in the RT-PCR assays was achieved using the “Gel-Pro Analyzer” software (Media Cybernetics). The transcript levels were normalized comparing the intensity of each mRNA signal to the γ-actin mRNA signal. Different cycles were tested in the RT-PCR in order to obtain optimal signals for quantitation. For the characterization of Pc16g04380 knock-down transformants, 40 cycles of amplification were used, whereas for the characterization of overexpression transformants, 36 cycles of amplification were employed. For γ-actin 36 cycles of amplification were used. Results were expressed as the mean and standard deviation of three measurements.

### 2.5. Sample Preparation for HPLC Analysis of Intracellular Levels of AdoMet, AdoHcy, Putrescine and Spermidine

Samples were taken at different time points from cultures in defined medium [30] with potassium phenylacetate (see above). Three different experiments were carried out in duplicate. Differences were considered as significant according to the standard deviation and when the p-value provided by the ANOVA test was *p* < 0.05. The mycelium was filtrated using a nylon filter (Nytal Maissa, Barcelona, Spain) and dried using Whatman filter paper. Then, the mycelium was ground to a fine powder in liquid nitrogen using a mortar and introduced into microtubes in order to determine the wet weight.

### 2.6. HPLC Analysis of Intracellular AdoMet and AdoHcy

AdoMet y AdoHcy were determined by HPLC protocols as previously reported [38,39]. Pulverized mycelia (see above) were resuspended in Milli Q water. The mobile phase consisted of 30 mM ammonium formate buffer pH 5.0 and acetonitrile (82:18). The flow rate was 1 mL/min. The run time was set to 30 min. Detection was carried out at 214 nm using an Agilent HPLC System equipped with a diode-array detector using an analytical 4.6 mm (inner diameter) × 250 mm (length) (5.0 μm particle size) RP C18 Lichrospher 100 (Merck) column.

### 2.7. HPLC Analysis of Intracellular Putrescine and Spermidine

Polyamines were detected after derivatization with benzoyl chloride, which provides more stable reaction products [40]. For this purpose, pulverized mycelia (see above) were resuspended in 1 mL of 2 M NaOH and 20 μL benzoyl chloride. The mixture was briefly shaken in a vortex and, after 20 min on ice, saturated NaCl solution (4.0 mL) and diethyl ether (4.0 mL) were added. This solution was mixed for 1 min and then centrifuged at 2000× *g* for 10 min. The upper ether phase containing benzoylated polyamines was transferred to another set of screw-capped tubes and evaporated to dryness. The residue was washed with diethyl ether and evaporated to dryness to remove any traces of water. This procedure was repeated two more times. Before injection and HPLC analysis, benzoylated polyamines were dissolved in 100 μL methanol and vortexed. After 10 min on ice, this methanol solution was filtered through Millipore filters (0.45 pm) to remove particulates [41]. Separation was carried out with gradient reversed-phase HPLC using an Agilent HPLC System equipped with a diode-array detector and using an analytical 4.6 mm (inner diameter) × 150 mm (length) (3.0 μm particle size) SPHERISORB C18 ODS (Waters) column. The mobile phase was a mixture of methanol-water (50:50, *v*/*v*). The flow rate was 0.8 mL/min. Polyamines were eluted with a gradient from 50% to 60% methanol (0 to 7 min) at room temperature. The gradient was returned to 50% methanol (10 to 11 min). The run time was set to 15 min. Detection was carried out at 240 nm.

### 2.8. HLPC Analysis of Benzylpenicillin Production

Samples were taken at different time points from cultures in defined medium [30] with potassium phenylacetate (see above). Analysis and quantitation of benzylpenicillin was carried out in culture supernatants by HPLC as previously described [34]. The specific production of benzylpenicillin (μg/mg dry weight) was assessed by dividing the volumetric production (μg/mL) into the dry weight (mg/mL). Three different experiments were carried out in duplicate. Differences were considered as significant according to the standard deviation and when the p-value provided by the ANOVA test was *p* < 0.05.

## 3. Results

### 3.1. P. chrysogenum Wisconsin 54-1255 Genome Contains a Putative AdoMet Synthetase-Encoding Gene, Which Is Constitutively Expressed under Normal and Penicillin-Producing Conditions

*In silico* analysis of the *P. chrysogenum* Wisconsin 54-1255 genome [42] revealed the presence of a single 1317-bp gene (Pc16g04380), whose cDNA (1164 bp) encodes a putative protein of 387 amino acids and a predicted molecular mass of 42.18 kDa (isoelectric point 5.66). The predicted amino acid sequence of this protein showed high similarity with well-characterized AdoMet synthetases from other eukaryotes, such as the trypanosomatid *Leishmania infantum* [21] (61% identity) or *Homo sapiens* [43] (68% identity), and with other filamentous fungi, such as AN1222 from *A. nidulans* [28] (90% identity) or PDE_07230 from *P. oxalicum* [29]. This protein contains the conserved motifs of AdoMet synthetases, namely the hexapeptide GAGDQG (responsible for binding adenine moiety of ATP) [44] between residues 123–128, and the glycine-rich nonapeptide GGGAFSGKD that has been proposed to bind the triphosphate moiety of ATP [45] between residues 270–278.

**Figure 2:**
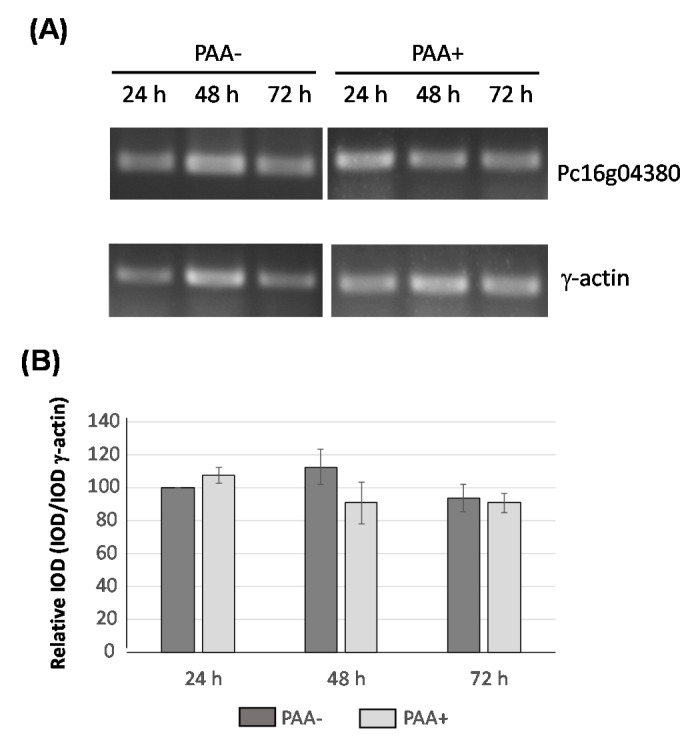
Transcriptional analysis of Pc16g04380 in the presence (PAA+) and absence (PAA-) of phenylacetic acid (PAA). (**A**) cDNA bands of Pc16g04380 and γ-actin were amplified by RT-PCR from RNA samples taken from cultures grown in complex medium during 24 h, 48 h and 72 h. (**B**) Integrated Optical Density (IOD) graph shows the expression profiles (normalized to the γ-actin expression levels) at 24 h, 48 h and 72 h. Those values corresponding to the expression of Pc16g04380 at 24 h were set to 100. Densitometry values correspond to the mean plus standard deviation of three independent measurements.

*In silico* and BLAST analyses of Pc16g04380 and the predicted encoded sequence revealed that this gene is present as a single copy in the genome. The Pc16g04380 gene is predicted to contain two introns (99 bp and 54 bp) close to the 5′-end region. Expression of this gene was tested in cultures of *P. chrysogenum* Wisconsin 54-1255, which was grown in complex medium in the presence and absence of phenylacetic acid during 72 h. RNA was extracted at different time-points and expression of the Pc16g04380 gene was confirmed by RT-PCR. As shown in Figure 2, expression is detected throughout the culture time and is not induced by phenylacetic acid.

### 3.2. Knocking-Down of the P. chrysogenum Pc16g04380 Gene Is Compatible with a Viable Phenotype

**Figure 3:**
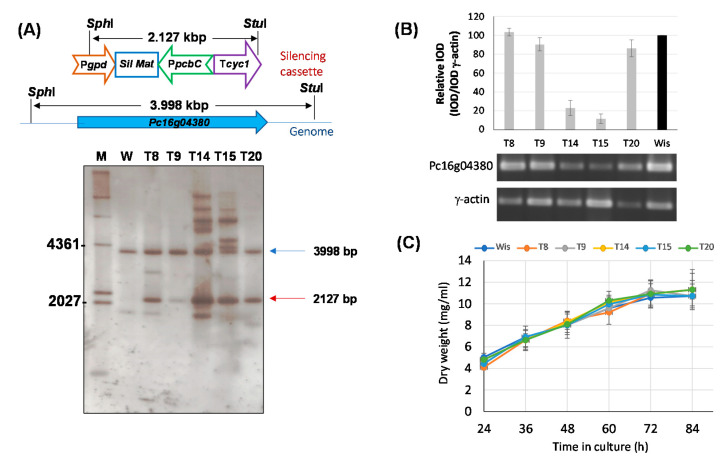
Silencing of the Pc16g04380 gene. (**A**) Southern blot analysis of different transformants and the parental *P. chrysogenum* Wisconsin 54-1255 strain (W) showing the integration of the 2127-bp silencing cassette (red arrow). The 3998-bp genomic band containing the genomic Pc16g04380 gene is indicated with a blue arrow. M (molecular weight marker). (**B**) Relative expression of Pc16g043801 in different transformants compared to the Wisconsin 54-1255. cDNA bands of Pc16g04380 and γ-actin were amplified by RT-PCR from RNA samples taken at 48 h from cultures grown in complex medium. Integrated Optical Density (IOD) graph shows the expression profile (normalized to the γ-actin expression levels) of each transformant regarding the values provided by the Wisconsin 54-1255 strain (Wis), which were set to 100. Densitometry values correspond to the mean ± SD of three independent measurements. (**C**) Growth profile in defined medium of Pc16g04380-knock-down transformants.

Functional characterization of the Pc16g04380 gene was initially tried using a knock-out approach. However, all attempts to obtain knock-out transformants were unsuccessful, thus suggesting that this gene is essential in *P. chrysogenum*. Therefore, in order to functionally characterize Pc16g04380, we carried out knock-down experiments to reduce the expression levels of this gene without compromising cell viability. For this purpose, protoplasts from *P. chrysogenum* Wisconsin 54-1255 were transformed with plasmid pJL43-RNAi-SAMsil, which includes a 467-bp exon fragment from the Pc16g04380 gene (see Materials and Methods). Several phleomycin-resistant transformants were obtained and analyzed by PCR to confirm the presence of the silencing cassette (data not shown). Five transformants that showed a correct amplification pattern were analyzed by Southern blotting after the digestion of genomic DNA with *Sph*I and *Stu*I (Figure 3A). The DIG-labelled exon fragment included in the silencing cassette was used as probe. All transformants and the parental strain showed the ~4-kbp band containing the genomic region with the Pc16g04380 gene. In addition to this band, transformants also included the ~2.1-kbp band from the silencing cassette. Transformants 14 and 15 showed additional hybridization bands, likely because of the ectopic random integration of partial fragments from the silencing cassette within the genome (Figure 3A).

In order to confirm Pc16g04380 gene silencing in transformants T8, T9, T14, T15 and T20, RT-PCR gene expression experiments were performed using RNA extracted at 48 h from cultures grown in complex medium without phenylacetic acid. As it can be inferred from Figure 3B, transformants T14 and T15 showed a 4.3- and 8.6-fold reduction, respectively, in the Pc16g04380 mRNA levels regarding those provided by the control strain. The rest of the transformants showed null (transformant T8) or very weak decrease in the transcription levels of Pc16g04380, ranging from 1.1-fold decrease (transformant T9) to 1.2-fold decrease (transformant T20).

So as to confirm whether knock-down transformants were impaired in growth, submerged cultures were carried out. All transformants grew well on solid Power medium and spore color did not differ from that of the parental strain (data not shown). For submerged cultures, we used defined medium to avoid the addition of components present in the complex medium that could mask the effect of a putative depletion of metabolites due to the knock-down of the Pc16g04380 gene. As seen in Figure 3C, all transformants showed a growth profile similar to that provided by the control strain, with similar amounts of dry weigh at each time point. This indicates that gene silencing of Pc16g04380 is compatible with a viable phenotype. Transformants T14 and T15 were selected for further experiments.

### 3.3. Pc16g04380 Encodes AdoMet Synthetase in P. chrysogenum (PcSasA)

**Figure 4:**
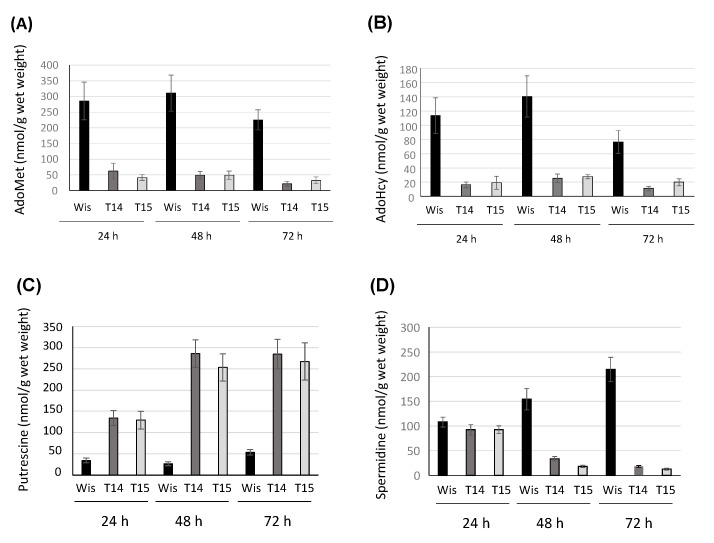
Specific production (nmol/g wet weight) of (**A**) AdoMet, (**B**) AdoHcy, (**C**) putrescine and (**D**) spermidine in the knock-down transformants T14 and T15 and the control strain *P. chrysogenum* Wisconsin 54-1255 (Wis). Results correspond to the mean ± SD from three different experiments carried out in duplicate.

In order to assess whether the protein encoded by Pc16g04380 is AdoMet synthetase, the intracellular levels of AdoMet and AdoHcy were analyzed by HPLC in knock-down transformants T14 and T15, and were compared to the levels provided by the control strain (*P. chrysogenum* Wisconsin 54-1255). As depicted in Figure 4A, knock-down transformants produced very low levels of AdoMet regarding the control strain. Significant differences ranged from a 4.6-fold (24 h) to a 10.5-fold (72 h) decrease in transformant T14, whereas for transformant T15, AdoMet levels remained around 6-fold lower than that provided by the control strain throughout the culture time.

AdoHcy production in the knock-down strain was also lower than in the control strain (Figure 4B). Differences ranged from a 5.5-fold (48 h) to a 7.0-fold (24 h) decrease in transformant T14, whereas in transformant T15, differences ranged from a 5.9-fold (24 h) to a 3.75-fold (72 h) decrease. These results confirm the role of the product of the Pc16g04380 in the biosynthesis of AdoMet, and in view of these results, Pc16g04380 was termed *PcsasA*.

Due to the role of AdoMet in polyamine biosynthesis, the intracellular levels of putrescine and spermidine were analysed by HPLC in knock-down transformants T14 and T15, and the control strain. Figure 4C shows that interestingly, putrescine levels increased in both transformants, which showed a similar behavior, regarding control values. The increase reached maximum values (~10 fold) at 48 h, whereas at 24 h and 72 h, values varied between a ~4-fold and a ~5-fold increase, respectively. On the other hand, spermidine values (Figure 4D) underwent a strong decrease at 48 h and 72 h in the knock-down transformants, with the lowest values (17-fold decrease) being reached by transformant T15 at 72 h.

### 3.4. Secondary Metabolism Is Affected by Reduced Levels of AdoMet

**Figure 5:**
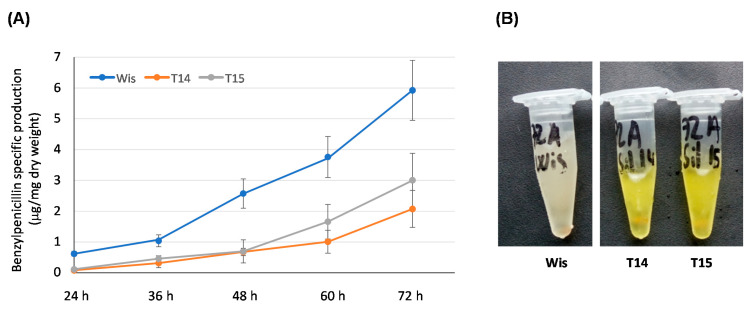
Secondary metabolism in knock-down transformants T14 and T15. (**A**) Benzylpenicillin specific production (μg/mg dry weight) of the control strain Wisconsin 54-1255 (Wis) and knock-down transformants T14 and T15 grown in defined medium. Results correspond to the mean ± SD from three different experiments carried out in duplicate. (**B**) Supernatants obtained after 72 h of growth from cultures of the control strain Wisconsin 54-1255 (Wis) and knock-down transformants T14 and T14 in defined medium. Note the yellow-orange color of the knock-down transformants.

The effect of AdoMet depletion on secondary metabolism was tested through benzylpenicillin production. With this purpose, cultures in defined medium were carried out and samples were extracted at different time points to analyze benzylpenicillin specific production (Figure 5A). Knock-down transformants T14 and T15 produced lower benzylpenicillin levels than the control strain. This significant decrease varied between 7.75-fold (24 h) and 3-fold (72 h) for transformant T14, and between 5.5-fold (24 h) and 2-fold (72 h) for transformant T15.

In addition to this effect on benzylpenicillin biosynthesis, knock-down transformants also accumulated a yellow-orange pigment in the culture broths (Figure 5B), which was more intense at 48 h and 72 h of culture. These results cannot conclude unequivocally that AdoMet levels act as a regulator of secondary metabolism, although it could be suggested that AdoMet is involved to some extent in processes related with secondary metabolism in *P. chrysogenum*.

### 3.5. Overexpression of PcsasA Does Not Lead to an Increase in Benzylpenicillin Production

**Figure 6:**
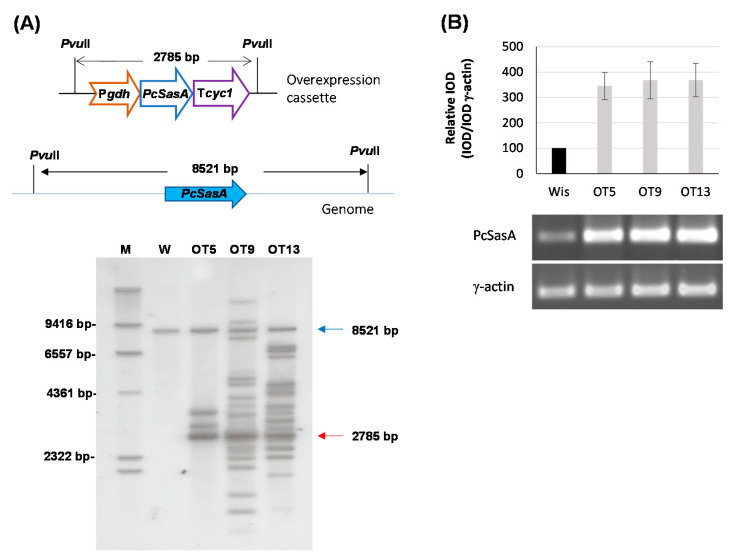
Overexpression of the Pc16g04380 (*PcsasA*) gene. (**A**) Southern blot analysis of different transformants and the parental *P. chrysogenum* Wisconsin 54-1255 strain (W) showing the integration of the 2785-bp overexpression cassette (red arrow). The 8521-bp genomic band containing the genomic Pc16g04380 (*PcsasA*) gene is indicated with a blue arrow. M (molecular weight marker). (**B**) Relative expression of Pc16g043801 (*PcsasA*) in different transformants compared to the Wisconsin 54-1255. cDNA bands of Pc16g04380 (*PcsasA*) and γ-actin were amplified by RT-PCR from RNA samples taken at 48 h from cultures grown in complex medium. Densitometry (IOD) graph shows the expression profile (normalized to the γ-actin expression levels) of each transformant regarding the values provided by the Wisconsin 54-1255 strain (Wis), which were set to 100. Densitometry values correspond to the mean ± SD of three independent measurements.

Since AdoMet levels are related to benzylpenicillin production, we decided to test whether overexpression of the *PcsasA* gene could lead to overproduction of this β-lactam antibiotic. Therefore, protoplasts obtained from *P. chrysogenum* Wisconsin 54-1255 were transformed with plasmid pOverSAM (see Materials and Methods), which includes the *PcsasA* gene under the control of the strong promoter of the *gdh* gene and the cyc1 transcriptional terminator. Transformants were selected in medium containing phleomycin and analyzed by PCR to confirm the presence of the overexpression cassette (data not shown). Three transformants that showed a correct amplification pattern were analyzed by Southern blotting after the digestion of genomic DNA with *Pvu*II (Figure 6A). The DIG-labelled exon fragment previously described was used as probe. All transformants and the parental strain showed the ~8.5-kbp band containing the genomic region with the *PcsasA* gene. In addition to this band, transformants also included the ~2.8-kbp band from the overexpression cassette. All transformants, especially OT9 and OT13, showed additional hybridization bands, which can be attributed to the ectopic random integration of partial fragments from the overexpression cassette within the genome (Figure 6A).

In order to confirm *PcsasA* overexpression, all transformants were tested by RT-PCR. With this purpose, cultures in complex medium without phenylacetic acid were conducted, and RNA was extracted from samples taken at 48 h. All transformants showed higher steady-state levels of PcSasA mRNA (around 3.5-fold increase) than the control strain (Figure 6B) and did not show sporulation or growth defects either in solid medium or in submerged cultures or differences in spore color regarding the parental strain (data not shown).

**Figure 7:**
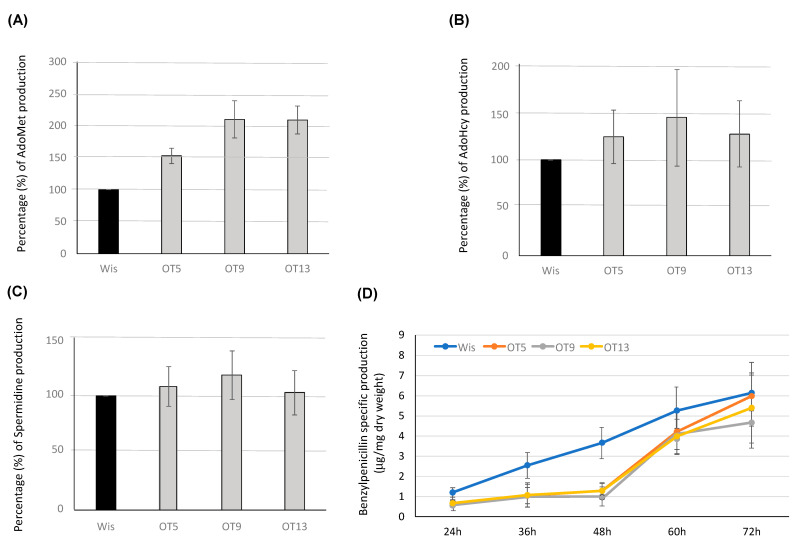
Production of AdoMet, AdoHcy, spermidine and benzylpenicillin in overexpression transformants. Percentage (%) of intracellular levels of (**A**) AdoMet, (**B**) AdoHcy and (**C**) Spermidine in the overexpression transformants OT5, OT9 and OT13 regarding values provided by the Wisconsin 54-1255 strain (Wis), which were set to 100%. (**D**) Benzylpenicillin specific production (μg/mg dry weight) of the control strain Wisconsin 54-1255 (Wis) and overexpression transformants OT5, OT9 and OT13 grown in defined medium. Results correspond to the mean ± SD from three different experiments carried out in duplicate.

Intracellular levels of AdoMet, AdoHcy and spermidine were quantified at 48 h in the parental and overexpression transformants. As seen in Figure 7A, transformants OT9 and OT13 were able to produce twice as many AdoMet as the parental strain. This significant increase was a bit lower in the case of transformant OT 5 (1.5-fold). When AdoHcy and spermidine levels were determined, no significant differences were found regarding the Wisconsin 54-1255 strain (Figure 7B,C).

Benzylpenicillin specific production was assessed in overexpression transformants (Figure 7D). Interestingly, antibiotic titers were not increased in these transformants. On the contrary, all transformants exhibited a significantly lower benzylpenicillin specific production than the Wisconsin 54-1255 strain during the first 48 h of culture. At this timepoint benzylpenicillin titers were approximately 3-fold lower in the overexpression transformants than in the control strain. After 48 h, β-lactam values in the overexpression transformants were not significantly different from those shown by the control strain, although they remained slightly below the control values with the only exception of transformants OT5, whose values reached those from the Wisconsin 54-1255 strain at the end of the culture time.

## 4. Discussion

AdoMet is the universal methyl donor molecule. This compound is synthesized by AdoMet synthetases, which are highly conserved proteins that in some organisms have one or more isoforms. In this work we have identified the gene encoding AdoMet synthetase of *P. chrysogenum* (PcSasA). *PcsasA* is present as a single copy in the *P. chrysogenum* genome, thus giving rise to only one form of the protein. The presence of a single AdoMet synthetase gene has also been reported in *P. oxalicum* and *A. nidulans* [28,29]. With the only exceptions of *Aspergillus flavus* and *Aspergillus oryzae*, which contain two independent isoforms of AdoMet synthetase, all *Aspergillus* species contain only one AdoMet synthetase encoding gene [28]. Similarly, *S. cerevisiae* also contains two independent AdoMet synthetase encoding genes (SAM1 and SAM2) in the genome [23,24].

AdoMet synthetases have been described as essential in fungi. Previous results confirmed that the gene encoding AdoMet synthetase in *A. nidulans* (*AnsasA*) is essential and its functions are indispensable for the cellular activities of this filamentous fungus [28]. Similar conclusions were raised in *P. oxalicum* [29]. Our initial attempts to obtain knock-out mutants in the Pc16g04380 gene were unsuccessful, which is consistent with the fact that this is gene encoding AdoMet synthetase (*PcsasA*) is essential for the viability of *P. chrysogenum* too.

We were able to reduce the steady-state levels of PcSasA mRNA by using a gene silencing approach, which resulted in a viable phenotype with normal growth and with lower levels of AdoMet and AdoHcy than the control strain. This produced accumulation of putrescine and reduction of spermidine due to likely reduction in dcAdoMet levels as a consequence of the depletion in AdoMet. Knocking-down of *PcsasA* gave rise to the production of lower penicillin titers than the control strain, which may be related to a decrease in spermidine levels. In *P. chrysogenum*, spermidine and 1,3-diaminopropane cause the reprogramming of metabolism, leading to multiple vesicles and penicillin overproduction [46]. This indicates that these compounds are also involved in controlling secondary metabolism in filamentous fungi. In fact, the intersection of the metabolisms of polyamines and β-lactams has also been recently confirmed in another β-lactam producer, *Acremonium chrysogenum*, where polyamines have been reported to upregulate cephalosporin C production and expression of β-lactam biosynthetic genes [47]. Interestingly, reduced levels of AdoMet also produced accumulation of a yellow-orange pigment. A similar effect was observed in *P. oxalicum*, where downregulated *PosasA* expression led to the formation of pink colonies instead of the dark-green colonies of the wild-type strain, which was attributed to the impairment of formation of melanins [29]. *P. chrysogenum* is able to synthesize several yellow pigments, such as chrysogine (chrysogenine) [48] and sorbicillinoids [49]. Identification of their biosynthetic pathways [50,51], together with other secondary metabolism enzymes related to the biosynthesis of natural products, pave the way to the engineering of *P. chrysogenum* in order to generate promising cell factories for novel products [52]. Overexpression of the *PcsasA* gene did not modify growth rate in *P. chrysogenum*. This agrees with the results shown after the overexpression of the genes encoding AdoMet synthetase in *N. crassa* or *P. oxalicum* [27,29], but it is in contrast with the results obtained in *A. nidulans*, which showed impaired development after overexpression of the *AnsasA* gene [28]. Increased expression of PcsasA gene led to the intracellular accumulation of AdoMet but did not significantly modify AdoHcy or spermidine levels. An explanation to this effect could be the sequestration of most of the excess AdoMet in vacuoles. Hence, vacuolar AdoMet would be excluded from the cytosolic pool of this molecule that is either decarboxylated (thus forming decarboxylated AdoMet) or demethylated (thus forming AdoHcy) as it has reported in *S. cerevisiae* [25]. *PcsasA* overexpression led to a reduction in benzylpenicillin levels along the culture, mainly at early time-points. A similar result was obtained in *A. nidulans* with sterigmatocystin, a precursor of aflatoxin B1. In this filamentous fungus, overexpression of *AnsasA* produced a decrease in the biosynthesis of this compound, which was attributed to defects in coordination of development and secondary metabolism [28]. Coordination of these two processes is regulated in filamentous fungi by the LaeA and Velvet Complex [9]. LaeA is a nuclear protein containing an AdoMet binding domain with putative methyltransferase activity and it has been hypothesized that it could regulate secondary metabolism by acting epigenetically through modification of chromatin structure, either directly or indirectly, although the exact function of this protein has not yet been clarified [53]. It has been suggested in *A. nidulans* that SasA could transiently interact with LaeA and the Velvet Complex and therefore, overproduction of SasA might affect LaeA function and secondary metabolite production [28]. In a similar way, the overproduction of PcSasA might interfere with LaeA and affect penicillin biosynthesis in *P. chrysogenum*. Future experiments will reveal if interaction occurs between these two proteins, which will help elucidate the mechanism of action of LaeA.

## Data Availability

Not applicable.
